# Inequality through the pipeline: racial and ethnic disparities remain in U.S. kidney transplantation

**DOI:** 10.1016/j.lana.2024.100924

**Published:** 2024-10-23

**Authors:** Laura C. Plantinga

**Affiliations:** Department of Medicine, University of California, San Francisco, 2540 23rd Street, Pride Hall Room 4403, San Francisco, 94110, CA, USA

Compared with dialysis treatment, kidney transplantation is associated with longer survival and better quality of life for patients^1^ and lower costs for society (*e.g*., per-person annual Medicare fee-for-service costs of $43,913 vs. $99,325 for transplantation vs. in–center hemodialysis^2^). Despite this, fewer than one-third (32%) of U.S. prevalent end-stage kidney disease (ESKD) patients have a transplant, with patients from minoritized populations being the least likely to have a kidney transplant (19%, 22%, 23%, 27%, and 34% of Native American, Native Hawaiian/Pacific Islander, Black, Hispanic, and Asian patients, respectively, vs. 38% of White patients). Given that the receipt of a kidney transplant is a complex process involving many steps, with multiple potential “leaks” in the “pipeline” (Fig. 1), knowing where in this pipeline —and among whom — disparities occur can help us target clinical and policy interventions to ensure greater equity in kidney transplantation. Clark-Cutaia et al.^3^ report on three steps of the pipeline (being informed of the kidney transplant option, placement on the waitlist, and receiving a kidney transplant) among patients treated with dialysis, using national administrative data. The researchers found that Black and Hispanic patients receiving dialysis were slightly more likely than White patients to be informed of the kidney transplant option at initiation but had less access to waitlisting and transplant; interestingly, compared to White patients, Asian patients were more likely to be waitlisted but less likely to be transplanted.^3^

**Fig. 1:**
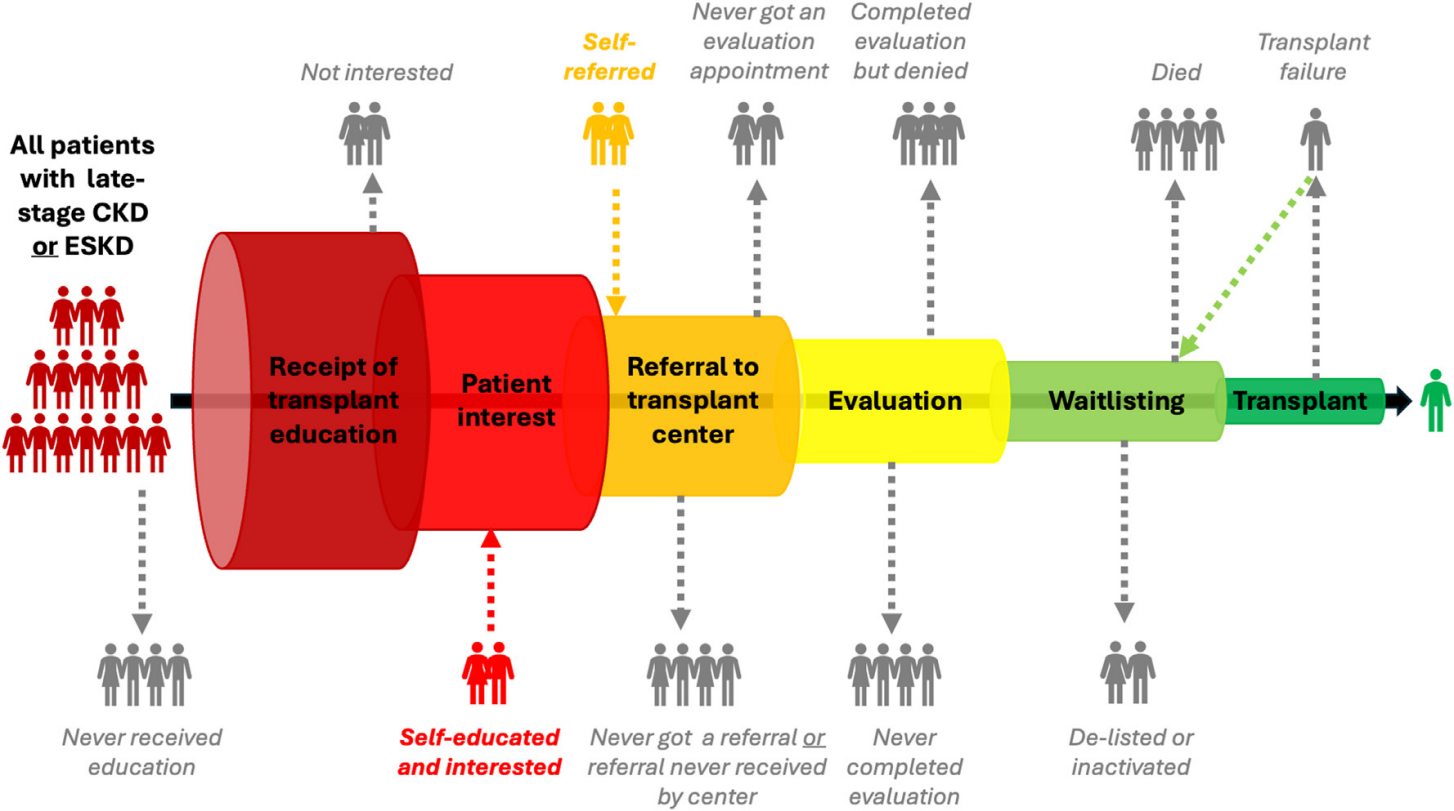
The U.S. kidney transplant “pipeline”. CKD, chronic kidney disease; ESKD, end-stage kidney disease.

These results contribute to our knowledge of inequities in the U.S. kidney transplant pipeline, while simultaneously highlighting important gaps that should be addressed in further research. First, although Cutaia et al.^3^ address three steps in the pipeline (Fig. 1), the same individuals were not studied at each step (*e.g*., transplant was evaluated among those who were waitlisted, not among those initiating dialysis). Studies examining the same individuals along the entire transplant pipeline are needed to understand the reasons for and impact of disparities at each step but are challenging with currently available data. Some steps in the pipeline may occur before dialysis initiation, when being informed of kidney transplant options was assessed in Clark-Cutaia et al.^3^; as well, there are steps between being informed and being waitlisted (Fig. 1) that are not available in national data. For example, regional data showed that White patients were actually less likely to be referred for kidney transplant than Black patients,^4^ similar to the results reported in Clark-Cutaia et al.^3^ regarding being informed of transplant options. While the finding that Black and Hispanic patients are more likely to be informed about the transplant option at dialysis initiation^3^ may partially reflect younger age and healthier status at onset, which are incompletely captured in administrative data, it may also reflect disparities in pre-emptive (*i.e*., prior to the onset of ESKD) referral, evaluation, waitlisting, or transplant.^5^^,^^6^ Thus, more studies identifying and targeting pre-ESKD transplant steps are warranted.

Additionally, many of the medical and, particularly, non-medical reasons [*i.e*., social determinants of health (SDOH)]^7^ for “leaks” in the transplant pipeline are not captured in national data. For example, the reasons why an individual who completes evaluations but is not waitlisted are not necessarily documented, and outcome letters sent to patients may not be transparent. These reasons likely differ by patient race and ethnicity due to race- and ethnicity-associated differences in SDOH, which may include: occupation, access to transportation, poverty, housing and food insecurity, language spoken, reading and health literacy, disability, and substance use. Also, these SDOH likely contribute to the extent to which patients self-educate and self-advocate, allowing them to continue through the pipeline, even without optimal support from their care team (Fig. 1).

In conclusion, Cutaia et al.^3^ highlight that disparities remain a substantial issue in kidney transplantation in the United States, despite policy changes targeted at later stages, such as the inclusion of waitlisting as a dialysis facility pay-for-performance measure^8^ and the changes in the Kidney Allocation System to mitigate racial disparities among those on the waitlist.^9^ This discrepancy may be partially due to the for-profit status of >80% of U.S. facilities, as these facilities are incentivized to address performance requirements but not to ensure that transplant education is of high quality or that patients complete necessary steps. The collection of national data on earlier steps in the transplantation pipeline,^10^ as well as more targeted studies on the effects of SDOH on pipeline leaks, would allow researchers and clinicians to understand and mitigate disparities throughout the kidney transplantation pipeline.

## Contributors

LCP was responsible for conceptualization, visualization, writing–original draft, and writing–review and editing.

## Declaration of interests

We declare no competing interests.
